# Retroperitoneal Extraovarian Fibrothecoma Mimicking a Malignant Epithelial Ovarian Carcinoma

**DOI:** 10.1155/2012/281745

**Published:** 2012-08-15

**Authors:** Patrick Roberts, Sharon Nofech-Mozes, Natalie Coburn, Paul Hamilton, Lilian T. Gien

**Affiliations:** ^1^Division of Surgical Oncology, Sunnybrook Health Sciences Centre, University of Toronto, Toronto, ON, Canada M4N 3M5; ^2^Division of Surgical Oncology, Princess Margaret Hospital, University of Toronto, Toronto, ON, Canada M5G 2M9; ^3^Department of Anatomical Pathology, Sunnybrook Health Sciences Centre, University of Toronto, Toronto, ON, Canada M4N 3M5; ^4^Sunnybrook Health Sciences Centre, Sunnybrook Research Institute, Toronto, ON, Canada M4N 3M5; ^5^Department of Health Policy, Management & Evaluation, University of Toronto, Toronto, ON, Canada; ^6^Division of Surgical Oncology, Sunnybrook Health Sciences Centre and Odette Cancer Centre, Toronto, ON, Canada M4N 3M5; ^7^Division of Radiology, Sunnybrook Health Sciences Centre, University of Toronto, Toronto, ON, Canada M4N 3M5; ^8^Division of Gynecologic Oncology, Sunnybrook Health Sciences Centre and Odette Cancer Centre, T2-104, 2075 Bayview Avenue, Toronto, ON, Canada M4N 3M5

## Abstract

*Background*. Fibrothecomas are benign sex cord-stromal tumors which rarely originate outside of the ovary. To date, two such cases have been reported in the literature. We report the third case of an extraovarian fibrothecoma and the first presenting similarly to a metastatic epithelial ovarian cancer. *Clinical History*. We describe a 62-year-old woman with history, physical examination, and imaging suggestive of metastatic ovarian cancer. CA-125 was elevated at 1291 U/mL. Paracenteses were negative for malignant cells and core biopsy showed spindle cell proliferation. A primary debulking surgery for a presumed ovarian cancer was planned. *Method and Results*. At surgery, 6 liters of ascites were drained. The uterus, ovaries, peritoneum, and omentum were normal. An 18 × 11 × 7 cm retroperitoneal mass was found between the left ureter and the sigmoid mesocolon, wrapped with sigmoid colon. Fallopian tubes and ovaries were normal. The mass was resected en bloc with the sigmoid colon, uterus, ovaries, and omentum. Microscopically, there was spindle cell proliferation typical of fibrothecoma. No ovarian tissue was identified in association with the tumor. *Conclusion*. This third case of extraovarian fibrothecoma highlights the importance of obtaining histologic evidence of malignancy prior to initiating neoadjuvant chemotherapy for a presumed ovarian cancer.

## 1. Introduction

Ovarian ectopia and neoplasms arising in them are extremely rare. To date, only two cases of extraovarian fibrothecoma have been reported. We report a third case of extraovarian fibrothecoma found in a patient with a presumed diagnosis of ovarian malignancy.

## 2. Clinical History

 A 62-year-old woman presented with a five-month history of increasing abdominal distention, weight loss, and an abdominal mass. There were no associated gastrointestinal symptoms or vaginal bleeding. On examination, she had gross abdominal distention and shifting dullness, consistent with ascites. There was no associated tenderness. The bowel sounds were normal to auscultation. Pelvic examination revealed a firm nodular mass with minimal mobility.

**Figure 1:**
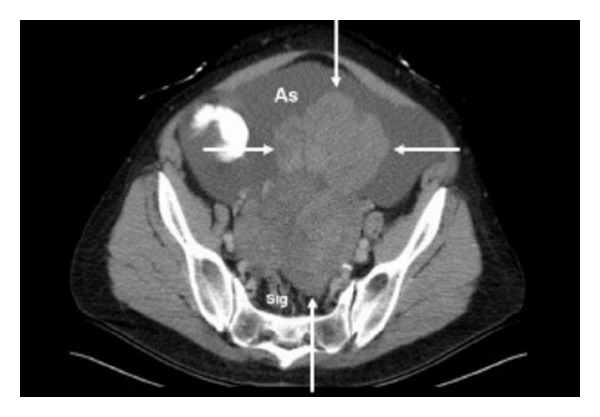
CT axial image of lower abdomen/pelvis demonstrating large predominantly solid mass (arrows) that appears to be arising from the retroperitoneum, surrounding sigmoid colon (Sig) and extending into peritoneal cavity. Mass is surrounded by a large amount of ascites (As).

 Abdominal and pelvic ultrasound demonstrated a large 15 cm complex and predominantly solid mass with cystic changes in the left adnexa. The right ovary was not seen, and the endometrium was not visualized. A moderate amount of ascites was also noticed around the liver, spleen, and lower quadrants. Computed tomography (CT) also showed a large, mainly solid, complex mass in the lower abdomen/pelvis displacing the uterus to the right side with extensive ascites (Figure 1). There was some suspicion of nodularity on the omentum, suggesting metastatic disease. A CT scan of the chest did not show any evidence of a pleural effusion. 

 Laboratory investigations revealed normal complete blood count, electrolytes and liver function tests. The serum CA125 was elevated at 1291 U/mL. Endometrial biopsy showed endometrial hyperplasia with atypia.

 Given the patient's clinical presentation, imaging findings, and elevated CA-125, there was a provisional diagnosis of metastatic epithelial ovarian cancer. The initial plan for management was to give neoadjuvant chemotherapy followed by interval debulking surgery. For both diagnostic and therapeutic purposes, four paracenteses were done to drain the ascites. Each paracentesis drained approximately 4 L of fluid. However, cytology of the peritoneal fluid was negative for malignant cells on all occasions. A CT-guided core biopsy of the mass was performed in order to get a pathologic diagnosis of malignancy prior to starting chemotherapy. The biopsy was performed in another institution and reported as spindle cell tumor negative for desmin, CD10, pankeratin, low-and high-molecular-weight keratin, CK7, CK20, S100, and CD34 and undetermined for CD117 immunohistochemistry. Ki-67 was immunoreactive in 5% of cells. The patient continued to be symptomatic with rapid accumulation of ascites. The inconclusive results on core biopsy and negative cytology led to the decision to proceed with a laparotomy and primary debulking surgery for a presumed ovarian cancer.

 At surgery, 6 L of ascites were immediately drained from the peritoneal cavity. Upon exploration of the abdomen, the ovaries and uterus were found to be completely normal. An 18 cm mass was found arising from the retroperitoneum between the left ureter and the mesentery of the sigmoid colon. A significant length of the sigmoid colon was wrapped around the mass. The omentum also looked normal and There was no evidence of peritoneal disease or enlarged para-aortic lymph nodes. An intraoperative frozen section of a portion of the retroperitoneal mass was done and showed spindle cells, with bland histology. The exact origin of the tumor was unclear intraoperatively. 

 The mass was resected en bloc with the sigmoid colon by a Hartmann's procedure. A total abdominal hysterectomy, bilateral salpingo-oophorectomy, and omentectomy were also performed. The postoperative course was complicated by an ileus and she was discharged on day eight. 

## 3. Method and Results

### 3.1. Gross Examination

**Figure 2:**
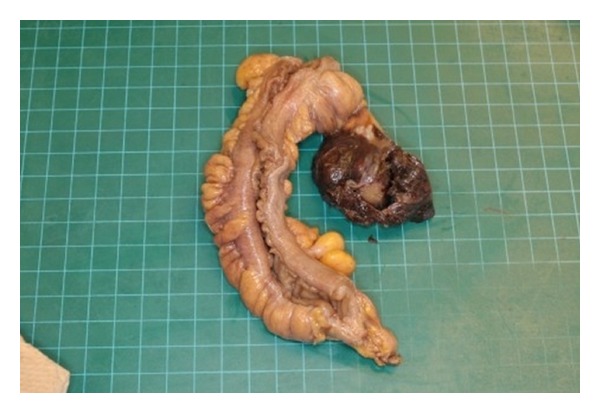
Macroscopic appearance of the tumor. The mass is attached to the mesorectum. (The ovaries, not shown, were grossly and microscopically unremarkable).

The main tumor mass measured 18 × 11 × 7 cm and consisted of a firm, solid, well-defined, and light brown lobulated mass with a homogeneous cut surface that was attached through the mesorectum to an 18 cm unremarkable segment of sigmoid rectum (Figure 2). A second 4 × 3 × 3 cm nodule with similar appearance was separately received for intraoperative consultation. The uterus showed thickened endometrium, a 2.3 × 1.5 × 0.8 sessile polypoid endometrial mass and subserosal leiomyomas. The attached fallopian tubes and ovaries were unremarkable. The ovaries measured 2.5 cm in their maximal dimension.

### 3.2. Microscopic Examination

**Figure 3:**
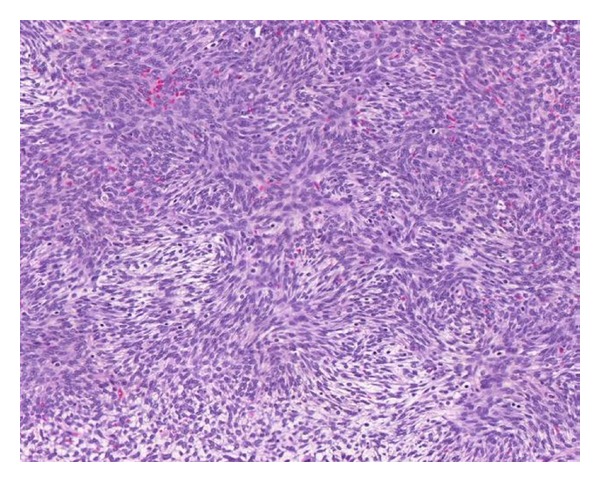
Representative section (hematoxylin and eosin ×40) illustrates cytologically bland fusiform cells arranged in whorls and fascicles alternating with more hypocellular areas with plumped cells with vacuolated cytoplasm.

 Twenty representative sections from the tumor were submitted. Those revealed a spindle cell proliferation typical of a fibrothecoma. The tumor consisted of fusiform cells arranged in whorls and fascicles alternating with more hypocellular areas with plumped cells (Figure 3). Focally, areas with hypocellular collagenous tissue typical of a fibroma were noted. Mitoses were infrequent (1-2 per 10 HPF). Nuclear grooves, cytologic atypia, and tumor necrosis were not identified. No ovarian tissue was identified in association with the tumor. The fallopian tubes and ovaries were entirely submitted for microscopic evaluation and showed no significant abnormality. Sections from the entirely submitted endometrium demonstrated an endometrial polyp and a spectrum of endometrial hyperplasia with areas of simple and complex hyperplasia with focal atypia.

### 3.3. Immunohistochemistry

**Figure 4:**
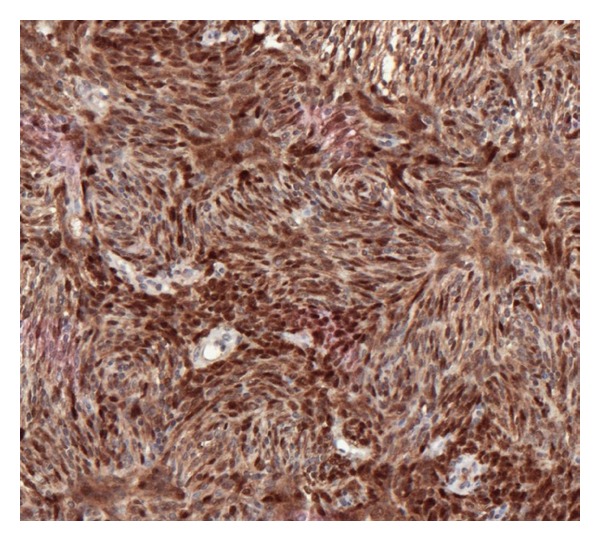
Immunohistochemistry—diffuse expression of calretinin.

**Figure 5:**
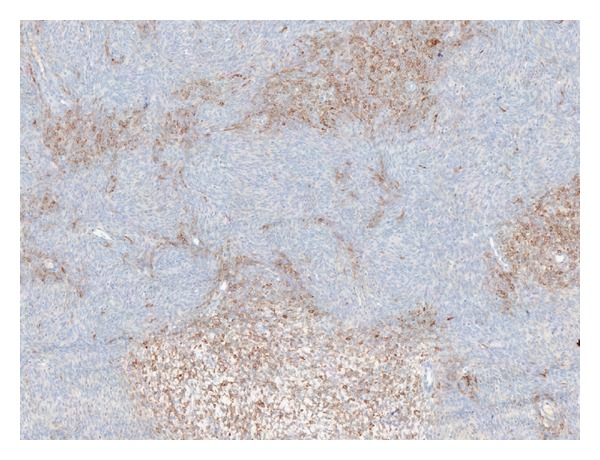
Immunohistochemistry—focal expression of inhibin.

The neoplastic cells were diffusely positive for vimentin, calretinin (Figure 4), CD99 and BCL2 and focally positive for inhibin (Figure 5).

The neoplastic cells were negative for the following markers: pankeratin and cytokeratin 5/6 (epithelial markers and mesothelial differentiation); CD34, CD117, and DOG1 (a panel used to rule out gastrointestinal stromal tumor); desmin, h-caldesmon and smooth muscle actin a (panel used to rule out smooth muscle proliferation); S-100 (a marker of neural and melanocytic differentiation); CD10 (a marker of endometrial stromal differentiation).

 The patient's ascites did not reaccumulate after surgery. The serum CA-125 was repeated a month after surgery and it normalized at 19 U/mL. At 3-month followup, the CA-125 was 7 U/mL, and CT scan of the abdomen and pelvis was negative for recurrence or metastasis. The stoma was reversed approximately 7 months after her initial surgery. The patient consented to the case report.

## 4. Discussion 

 Fibrothecomas constitute only 4% of all ovarian tumors and are the most common solid tumors of the ovary [1–3]. Tumors of the ovary can rarely develop in extraovarian and pelvic locations, presumably arising in ectopic ovarian tissue. So far only two extraovarian fibrothecomas have been reported in the literature [4, 5], and there have been two reports of other types of benign extraovarian gonadal stromal cell tumors (extraovarian thecoma and fibroma) [6, 7], presenting in the broad ligament or cul-de-sac. Some reports discuss sex cord-stromal tumors, such as granulosa cell tumors or Sertoli-Leydig tumors, presenting in extraovarian locations [7, 8]; however, the current paper is one of the few to demonstrate a benign entity presenting in the retroperitoneum mimicking a malignancy [9].

 The etiology of how a fibrothecoma develops in an extraovarian location is unknown and its histogenesis remains speculative. There are two types of heterotopic ovarian tissue: (1) the accessory ovary, which is connected to, or lies close to, the orthotopic ovary and arises from the same primordium; (2) the supernumerary ovary, which is a third ovary located at a distance from the bed and arises from a separate anlage [4]. Most supernumerary ovaries are incidental findings at autopsy, laparoscopy, or surgery. Rare cases of documented pathology in supernumerary ovaries have been reported [10, 11]. Other possibilities include derivation from Mullerian rests or from the mesothelium [12]. Regardless of their origin, these extraovarian fibrothecomas are histologically indistinguishable from their ovarian counterparts. 

 This is the first reported case of an extraovarian fibrothecoma presenting with symptoms and findings similar to a metastatic epithelial ovarian cancer with gross ascites and an elevated CA-125. Ovarian fibrothecomas can be associated with Meigs' syndrome, typically described as the triad of ascites, unilateral hydrothorax, and benign ovarian tumor [13]. Meigs' syndrome resolves with the removal of the benign ovarian mass. In this case, the patient had continual reaccumulation of ascites which resolved once the pelvic tumor was removed, similar to that expected for an ovarian fibrothecoma. This patient also presented with a significantly elevated CA-125. Serum CA-125 levels are elevated in 80–85% of patients with epithelial ovarian cancers [14]. However, CA-125 is a nonspecific test that can also be elevated in a number of nonmalignant conditions, including liver disease, pelvic inflammatory disease, benign fibroids, or pregnancy. CA-125 is expressed by mesothelial cells of the serosal membrane in pleura, pericardium, and peritoneum, and its production is increased with inflammation of these structures [15]. The presence of ascites, such as in Meigs' syndrome, can cause inflammation of the peritoneum and subsequently elevate the serum CA-125 [14–16]. This case illustrates the importance of considering a differential diagnosis when a patient presents with ascites and an elevated CA-125.

 The diagnosis of extraovarian fibrothecoma preoperatively would be greatly assisted if characteristic imaging findings existed. The two previously reported cases of extraovarian fibrothecoma were in the 1980s, before the regular usage of modern day imaging modalities. Although sonography has been considered as the imaging modality of choice in detection of ovarian masses, the sonographic features of ovarian fibrothecoma are nonspecific [1]. These tumors usually present as solid, hypoechoic masses with strong attenuation on ultrasound. Other adnexal masses, including both primary and secondary tumors such as metastasis, granulosa cell tumor, and pedunculated uterine myomas, can also present as solid, hypoechoic masses [1]. On CT scans, these tumors have been described as solid masses with delayed accumulation of contrast medium. This morphologic appearance may be affected by intratumoral hemorrhage, infarction, or cystic degeneration [1]. Pelvic MRI was not performed in this case. Fibrous tumors such as ovarian fibromas, fibrothecomas, and uterine leiomyomas will typically demonstrate low signal on T2-weighted sequences, whereas most other solid neoplasms would be high signal on T2-weighted sequences. We would expect an extraovarian fibrothecoma to have similar low-signal T2-weighted characteristics. However, again, this would be a nonspecific finding and given the rarity of an extraovarian fibrothecoma, it would unlikely be diagnostic.

 Due to the rarity of extraovarian fibrothecoma and the difficulties with making the diagnosis preoperatively, the diagnosis will likely continue to be made after surgery. Ovarian fibrothecomas rarely pose a diagnostic challenge, and designation as extraovarian fibrothecoma can be made only when normal ovaries are confirmed. Preoperatively when diagnostic material is limited to either cytology or core biopsy, reaching a definitive diagnosis is more difficult. Sex cord-stromal tumors should be included in the differential diagnosis of retroperitoneal mass with ascites, and a panel of immunohistochemical markers such as calretinin, CD99, and inhibin can be used as an adjunct diagnostic tool. The sensitivity for sex cord-stromal lineage may vary between markers, and some markers may not be as sensitive in some types of sex cord-stromal tumors compared with other tumors in this spectrum of neoplasms [17, 18]. These markers are not specific and do not discriminate between various tumors within the sex cord-stromal category. The final classification is based on morphology. Mitoses are commonly seen in fibrothecoma therefore finding of 5% ki67 immunoreactivity as reported on the preoperative core biopsy does not preclude the diagnosis [19]. 

 Although ovarian fibrothecomas are not unusual, this case represents the third reported case in the literature of an extraovarian fibrothecoma. It is also the first reported extraovarian fibrothecoma to present with symptoms and findings similar to that of metastatic ovarian cancer. This case highlights the importance of obtaining cytologic or pathologic evidence of malignancy prior to initiating neoadjuvant chemotherapy for a presumed ovarian cancer. Despite the benign diagnosis in this case, radical surgery would still be required to remove the tumor.
